# Serum cotinine cut-points for secondhand smoke exposure assessment in children under 5 years: A systemic review

**DOI:** 10.1371/journal.pone.0267319

**Published:** 2022-05-05

**Authors:** Nerea Mourino, Alberto Ruano-Raviña, Leonor Varela Lema, Esteve Fernández, María José López, María Isolina Santiago-Pérez, Julia Rey-Brandariz, Alexandra Giraldo-Osorio, Mónica Pérez-Ríos

**Affiliations:** 1 Department of Preventive Medicine and Public Health, University of Santiago de Compostela, Santiago de Compostela, Spain; 2 CIBER Epidemiology and Public Health, CIBERESP, Madrid, Spain; 3 Tobacco Control Unit, WHO Collaborating Center for Tobacco Control, Institut Català d’Oncologia (ICO), Badalona, Spain; 4 Tobacco Control Research Group, Institut d’Investigació Biomèdica de Bellvitge (IDIBELL), Barcelona, Spain; 5 School of Medicine and Health Sciences, Universitat de Barcelona, Barcelona, Spain; 6 CIBER of Respiratory Diseases (CIBERES), Madrid, Spain; 7 Servicio de Evaluación y Métodos de Intervención, Agència de Salut Pública de Barcelona, Barcelona, Spain; 8 Institut d’Investigació Biomèdica de Sant Pau (IIB Sant Pau), Barcelona, Spain; 9 Epidemiology Unit, Galician Directorate for Public Health, Santiago de Compostela, Spain; 10 Departamento de Salud Pública, Grupo de investigación Promoción de la Salud y Prevención de la Enfermedad (GIPSPE), Universidad de Caldas, Manizales, Colombia; 11 Fundación Carolina, Madrid, Spain; Holbaek Sygehus, DENMARK

## Abstract

**Background:**

Serum cotinine has become the most widely used biomarker of secondhand smoke exposure (SHS) over time in all ages. The aim of this study was to review the serum cotinine cut-points used to classify children under 5 years as exposed to SHS.

**Methods:**

A systematic review performed in the Pubmed (MEDLINE) and EMBASE databases up to April 2021 was conducted using as key words "serum cotinine", “tobacco smoke pollution” (MeSH), "secondhand smoke", "environmental tobacco smoke" and “tobacco smoke exposure”. Papers which assessed SHS exposure among children younger than 5 years old were included. The PRISMA 2020 guidelines were followed. Analysis was pre-registered in PROSPERO (registration number: CRD42021251263).

**Results:**

247 articles were identified and 51 fulfilled inclusion criteria. The selected studies were published between 1985–2020. Most of them included adolescents and adults. Only three assessed postnatal exposure exclusively among children under 5 years. None of the selected studies proposed age-specific cut-points for children < 5 years old. Cut-point values to assess SHS exposure ranged from 0.015 to 100 ng/ml. The most commonly used cut-point was 0.05 ng/ml, derived from the assay limit of detection used by the National Health and Nutrition Examination Survey (NHANES).

**Conclusions:**

No studies have calculated serum cotinine age-specific cut-points to ascertained SHS exposure among children under 5 years old. Children’s age-specific cut-points are warranted for health research and public health purposes aimed at accurately estimating the prevalence of SHS exposure and attributable burden of disease to such exposure, and at reinforcing 100% smoke-free policies worldwide, both in homes, private vehicles and public places.

## Introduction

The assessment of the involuntary smoking or exposure to secondhand smoke (SHS) in children during pregnancy and early childhood is essential for public health and research purposes. Estimating the children at-risk from environmental tobacco can have important public health implications in terms of determining the population attributable burden of disease and evaluating the impact of the information, cessation interventions and prohibition laws on this target group. SHS is defined as tobacco smoke produced by an active smoker from exhaled mainstream smoke together with sidestream smoke. Sidestream smoke is a combination of smoke from the smoldering tobacco product between and during puffs and smoke components which diffuse through cigarette paper [1,2].

Pediatric population is especially vulnerable to the effects of SHS due to their narrower bronchi, faster respiratory rate and immature immune system [3]. Prejudicial effects derived from SHS exposure on children’s health have been documented since the 1970s, including but not limited to the following: increased risk of sudden infant death syndrome, acute respiratory symptoms (cough, phlegm, wheeze and breathlessness) and ear infections [1]. In 2004, 165,071 deaths among children younger than 5 years were attributed worldwide to SHS exposed [4]. However, these estimations were considered to be prone to bias because exposure data was mainly derived from surveys. Parental self-report questionnaires have been widely used to assess both prenatal and postnatal SHS exposure [5,6] but frequently provide underestimations due to parents’ reluctance to disclose their smoking status and underreporting of children’s SHS exposure, aswell as the recall bias or lack of knowledge of exposure [6,7]. This highlights the need for objective methods based on the determination of biomarkers to provide reliable assessment of SHS exposure [5].

Cotinine, the major metabolite of nicotine, is considered the best biomarker for assessing recent SHS exposure due to its high specificity and sensitivity, as well as its relatively prolonged half-life which ranges from 16 to 20 hours in children [6,8]. Cotinine can be collected from various biological samples such as blood (serum/plasma), urine, saliva, hair, meconium and maternal milk, and quantified through several analytical techniques [6,8–10]. Although the collection of serum or plasma cotinine is more invasive than that from other samples, it is not influenced by renal function, flow rate, and urinary pH and it does not require urine dilution adjustments; over time, serum cotinine has become the most widely used biomarker of SHS exposure among children [1,6].

Serum cotinine allows for differentiating active smokers from passive and non-smokers. Nonetheless, the determination of SHS exposure is rather challenging as currently there is no standardized consensus regarding the optimal cut-point to be used to classify SHS exposure among young children. Some prior studies, based on NHANES data for older children and adolescents, proposed the serum cotinine cut-point of 0.05 ng/ml which derives from the assay limit of detection (LOD) [8,11]. This cut- point maximises sensitivity to avoid missing positive cases, but it should be acknowledged that a trade-off always exists between sensitivity and specificity, and cotinine metabolism and clearance can vary by sex, race and age [1,5]. Considering that children´s physiology is quite different within the 0–5 years old age range, age-specific cut-points might be useful for different studies to accurately classify nonsmoking children as exposed or unexposed to tobacco smoke in a standardized manner [5]. As far as we are aware of, there are no reviews focused on the serum cotinine cut-points used to classify SHS exposure in the pediatric population.

The aim of this study is to review serum cotinine cut-points used so far to assess SHS exposure in children under 5 years and assess the changes on the cut-point values over time and across different countries.

## Material and methods

### Literature search

We performed a systematic review in PubMed (MEDLINE) and EMBASE databases following the PRISMA 2020 guidelines [12]. Search included papers published until April 2021 and “serum cotinine”, “Tobacco smoke pollution” (MeSH), “secondhand smoke” and “environmental tobacco smoke” were used as key words. Search strategy underwent peer review by three knowledgeable reviewers. Age filters were set (newborns-infant-preschool child). No reports, communications to congresses or simulation essays were included. The bibliographic search was limited to English, Spanish, French and German. Analysis was pre-registered in PROSPERO (registration number: CRD42021251263).

### Inclusion/Exclusion criteria

To be selected for this review, articles had to assess pre-natal SHS exposure in newborns and/or post-natal SHS exposure in children younger than 5 years by using serum cotinine assays in addition to or in lieu of parental self-report. We included investigations with mother-child pairs and those grouping children under 5 with older ones, adolescents and adults. Among the latter, when data was stratified by age groups, we selected that including children younger than 5 years. On the other hand, we excluded studies with serum cotinine obtained just from mothers; without cut-point values to distinguish between exposed and unexposed children, and those measuring cotinine from other biological fluids as urine, saliva, hair, meconium or maternal milk. In the case of longitudinal studies, we only considered the information corresponding to the period of time in which SHS exposure was ascertained accompanied with serum cotinine in a sample including children under 5 years.

### Selection of articles and evidence synthesis

After excluding duplicate articles from the search, the title and abstract of the records were screened individually by two independent reviewers to ascertain that they met eligibility criteria. Full text of the eligible papers was obtained for potentially relevant articles. Bibliography from the selected papers was revised to manually complete the initial search. Thereafter, data from those meeting eligibility criteria were extracted and discrepancies in its interpretation were solved by consensus. A modified Newcastle-Ottawa scale was used to assess appropriateness of the representativeness of the sample, participation rate, ascertainment of the exposure, and the adequacy of the method used for the selection of the cut-points. Score disagreements were resolved by consensus and a final agreed-upon rating was assigned to each study.

The data extracted for analysis were: study characteristics (author, publication year, design, period of the study, use of questionnaire in conjunction with cotinine (yes/no) and country); population characteristics (sample size, age and race); information regarding the analytical technique used to quantify serum cotinine (radioimmunoassay (RIA), enzyme-linked immunosorbent essay (ELISA), gas chromatography (GC), gas-chromatography mass spectrometry (GC/MS), gas-liquid chromatographic procedure with nitrogen-phosphorus-specific detector (GLC/NPD) and isotope dilution liquid chromatography tandem mass spectrometry (LC-MS)); serum cotinine cut-point values to classify SHS exposure in children and method for the selection of the cut-points.

## Results

**Fig 1:**
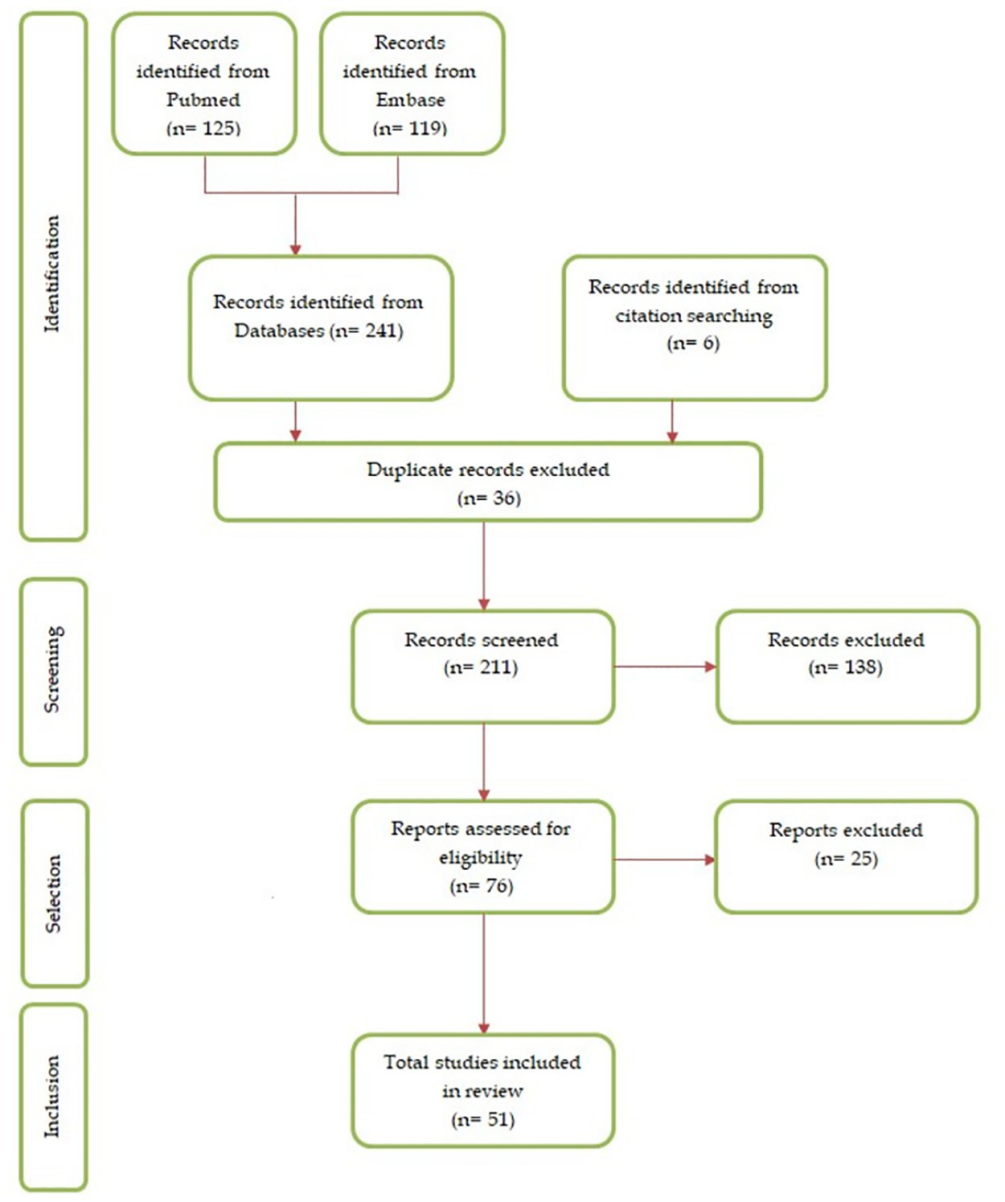
PRISMA 2020 flow diagram for studies selection (search until April 2021).

A total of 247 articles were obtained and 51 of them fulfilled the inclusion criteria. The PRISMA flow diagram of the included studies is displayed in Fig 1.

### Study and population characteristics

The 51 papers included were published between 1985 and 2020. They included one case-control study [13], 13 cohort studies [14–26] and 37 [27–63] cross-sectional studies from which, one included a matched case-control substudy [53]; 26 studies obtained data from different cycles of the National Health and Nutrition Examination Survey (NHANES). When applying the modified Newcastle-Ottawa scale, two cohort studies [17,19] and one cross-sectional study [36], which used data from the NHANES, were judged to be of high quality, and thirty six studies were judged to be of low quality.

**Fig 2:**
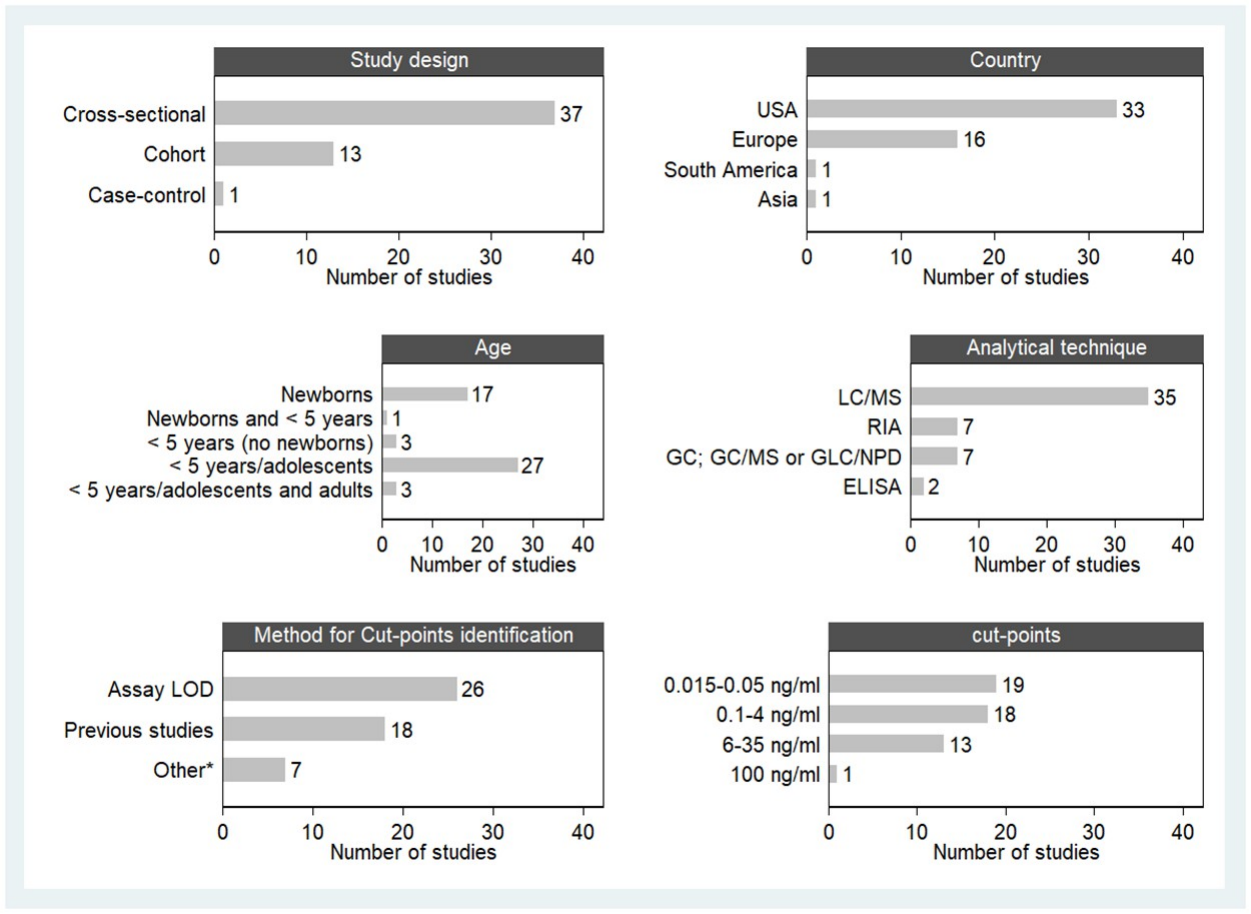
Study and population characteristics (design, country; age); analytical technique for serum cotinine quantification (isotope dilution-liquid chromatography-tandem mass spectrometry (LC/MS), radioimmunoassay (RIA), gas chromatography (GC), gas-chromatography mass spectrometry (GC/MS), gas-liquid chromatographic procedure with nitrogen-phosphorus-specific detector (GLC/NPD) and enzyme-linked immunosorbent essay (ELISA)); cut-point values range (ng/ml) and methods for cut-point identification (assay limit of detection (LOD), previous studies or other (separation point in the bimodal distribution, median cotinine levels of the cohort, receiver operating characteristic (ROC) curves or percentiles)).

Studies were mainly conducted in the USA (34 papers) and Europe (15 studies) (Fig 2).

Thirty-three out of 51 papers measured postnatal exposure to SHS with serum cotinine; 17 prenatal exposure with newborns’cord serum cotinine and one both pre- and post-natal exposure [19]. 38 studies ascertained exposure by using both serum cotinine and questionnaires (Table 1).

**Table 1:** Study and population characteristics; analytical technique for serum cotinine quantification; cotinine cut-points (CP) and method for their selection.

STUDY CHARACTERISTICS				POPULATION CHARACTERISTICS			ANALYTICAL TECHNIQUE, CP, METHOD FOR THE SELECTION OF THE CP AND REFERENCE NUMBER			
Author, yr	Design and period	Questionnaire (yes/no)	Country	Sample size	Age	Race	Analytical technique	CP (ng/ml)	Method Reference	
Luck et al, 1985 [27]	Cross-sectional, N/A	Yes	Germany	8	Newborns	N/A	GLC/NPD	**5**	Assay LOD	27
Ahlsten et al, 1989 [28]	Cross-sectional, N/A	Yes	Sweden	39	Newborns	N/A	GC	**1**	Assay LOD	28
Etzel et al, 1992 [14]	Cohort study, 1964–1983	No	USA	132	4 months-3 yr	White, black or mixed race	RIA	**2.5**	ROC curves	14
Bardy et al, 1993 [31]	Cross-sectional, 1991	Yes	Finland	1,237	Newborns	N/A	GC/MS	**6**	Assay LOD	31
CDC,1993 [29]	Cross-sectional (NHANES, 1988–1991)	Yes	USA	800†	4–91 yr	N/A	LC/MS	**10/15**	Authors selected the CP value from a separation point in the bimodal distribution of SC in smokers and nonsmokers	29
Bardy et al, 1994 [30]	Cross-sectional, 1991	Yes	Finland	1,323	Newborns	N/A	GC/MS	**35**	Assay LOD	30
Martinez et al, 1994 [15]	Cohort Study, 1980–1984	Yes	USA	175	Newborns	Hispanic or other non-Anglo	RIA	**1**	Assay LOD	15
Ruhle et al, 1995 [32]	Cross-sectional, N/A	Yes	Germany	75	Newborns	N/A	RIA	**15**	Authors selected the CP value based on data from a previous study	32
Pirkle et al, 1996 [33]	Cross-sectional (NHANES, 1988–1991)	Yes	USA	1,793‡	4–11 yr	Non-Hispanic white, non-Hispanic black, Mexican American or other	LC/MS	**10/15**	Authors selected the CP value from a separation point in the bimodal distribution of SC in smokers and nonsmokers	33
Nafstad et al, 1996 [16]	Cohort study, 1992–1993	Yes	Norway	202	Newborns	N/A	RIA	**14**	Authors selected the CP value based on data from a previous study	16
Bearer et al, 1997 [13]	Case-control, 1991–1992	Yes	USA	70	Newborns	Non-Hispanic white, non-Hispanic black or other (Hispanic and Asian)	GC/MS	**1**	Assay LOD	13
Pichini et al, 2000 [17]	Cohort Study, 1997–1998	Yes	Spain	429	Newborns	N/A	RIA	**1,78**	ROC curves	17
Mannino et al, 2001 [34]	Cross-sectional (NHANES, 1988–1994)	Yes	USA	1,533	4–6 yr	Non-Hispanic white, non-Hispanic black or Mexican American	LC/MS	**0.05**	Assay LOD	34
Strauss, 2001 [35]	Cross-sectional (NHANES, 1988–1991)	Yes	USA	2,968‡	4–18 yr	N/A	LC/MS	**2**	Author selected the CP value based on data from a previous study	35
Mannino et al, 2001 [36]	Cross-sectional (NHANES, 1989–1994)	Yes	USA	5,653‡	4–16 yr	Non-Hispanic white, non-Hispanic black or Mexican American	LC/MS	**0.05**	Assay LOD	36
Lieu et al, 2002 [37]	Cross-sectional (NHANES, 1989–1994)	Yes	USA	1,825‡	4–12 yr	Non-Hispanic white, non-Hispanic black or Mexican American	LC/MS	**10/20**	Authors selected the CP value based on data from a previous study	37
Mannino et al, 2002 [38]	Cross-sectional (NHANES, 1989–1994)	No	USA	523‡	4–16 yr	Non-Hispanic white, non-Hispanic black or Mexican American	LC/MS	**0.05**	Assay LOD	38
Rubin et al, 2004 [39]	Cross-sectional (NHANES, 1989–1994)	Yes	USA	6,153‡	4–16 yr	N/A	LC/MS	**2.9**	Authors selected both CP values based on data from a previous study	39
Aligne et al, 2003 [40]	Cross-sectional (NHANES, 1989–1994)	No	USA	3,531‡	4–11 yr	Non-Hispanic white, non-Hispanic black, Mexican American or other	LC/MS	**0.05**	Assay LOD	40
Wilkinson et al,2006 [41]	Cross-sectional (NHANES, 1989–1991)	Yes	USA	2,516‡	4–16 yr	Non-Hispanic white, non-Hispanic black, Mexican American or other	LC/MS	**0.05**	Assay LOD	41
Pirkle et al, 2006 [42]	Cross-sectional (NHANES, 1989–2002)	Yes	USA	1988–91: 1,839‡ 1991–94: 2,090‡ 1999–2000: 1,065‡ 2001–2002: 1,278‡	4–11 yr	Non-Hispanic white, non-Hispanic black, Mexican American or other	LC/MS	**0.05** **0.015**	Assay LOD	42
Franchini et al, 2008 [43]	Cross-sectional, 2004–2005	Yes	Italy	979	Newborns	Italian or foreign	GC/MS	**1**	Authors selected the CP value based on data from previous studies	43
Puig et al, 2008 [18]	Cohort Study, 1996–1998.	Yes	Spain	487	Newborns	Spanish or non-Spanish	RIA	**1**	Authos selected the CP value based on data from a previous study	18
Max et al, 2009 [44]	Cross-sectional (NHANES, 1999–2006)	Yes	USA	1999–2000: 1,179‡ 2001–2002: 1,423‡ 2003–2004: 1,265‡ 2005–2006: 1,300‡	3–11 yr	Non-Hispanic white, non-Hispanic black, Mexican American, other Hispanic or other race/ethnicity	LC/MS	**0.05** **0.015**	Assay LOD	44
Dixon et al, 2009 [29]	Cross-sectional (NHANES, 1999–2004)	Yes	USA	829‡	1–5 yr	Non-Hispanic white, non-Hispanic black, Hispanic or other	LC/MS	**0.05** **0.015**	Assay LOD	45
CDC (Vital signs), 2010 [46]	Cross-sectional (NHANES, 1999–2008)	Yes	USA	N/A	3–11 yr	Non-Hispanic white, non-Hispanic black, Mexican American or other	LC/MS	**0.05**	Assay LOD	46
Dove et al, 2010 [47]	Cross-sectional (NHANES, 1999–2006)	Yes	USA	1,582‡	3–5 yr	Non-Hispanic white, non-Hispanic black, Mexican American or other	LC/MS	**0.05**	Assay LOD	47
Vesper et al, 2010 [48]	Cross-sectional (NHANES, 2003–2004)	No	USA	N/A	3–11 yr	Non-Hispanic white, non-Hispanic black or Mexican American	LC/MS	**10**	Authors selected the CP value based on data from a previous study	48
Xu et al, 2010 [49]	Cross-sectional (NHANES, 2001–2002)	No	USA	4,508‡	4–15 yr	Non-Hispanic white, non-Hispanic black, Mexican American or other	LC/MS	**0.035**	Authors selected CP values based on the 33rd and 67th percentiles of the SC concentrations	49
Preston et al, 2010 [50]	Cross-sectional, 2004–2005	Yes	USA	30	Newborns	Asian, Caucasian or African American	LC/MS	**1**	Authors selected the CP value based on data from a previous study	50
Sharief et al, 2011 [51]	Cross-sectional (NHANES, 2005–2006)	No	USA	3,136†	1–21 yr	Non-Hispanic white, non-Hispanic black, Mexican American or other	LC/MS	**2.9**	Authors selected the CP value based on data from a previous study	51
Spanier et al, 2011 [19]	Cohort Study, 2003–2006	Yes	USA	Newborns: 273 1 yr: 275 2 yr: 206	Newborns-2 yr	Non-Hispanic White, non-Hispanic Black or other	LC/MS	**0.015**	Assay LOD	19
Cardwell et al, 2012 [52]	Cross-sectional, N/A	No	USA	220‡	1–16 yr	N/A	LC/MS	**0.9** **0.6**	ROC curves; 0.9 ng/ml for children aged < 12 yr; 0.6 ng/ml for those aged ≥12 yr.	52
Dempsey et al, 2012 [53]	Cross-sectional with a matched case control substudy, 2009–2010	Yes	USA	274‡	8 months- 17 yr (70% were < 3 yr)	Latino, African American, Asian, White non-Hispanic or other	LC/MS	**0.05**	Assay LOD	53
Florath et al, 2014 [20]	Cohort Study, 2000–2001	Yes	Germany	972	Newborns	German or non-German	LC/MS	**14**	Authors selected the CP value based on data from previous studies	20
Wang et al, 2013 [21]	Cohort study	Yes	China	14	Newborns	N/A	LC/MS	**0,12**	Authors selected the CP value based on the median cotinine levels of the entire cohort	21
Andersen et al, 2013 [54]	Cross-sectional, 1988–1990	No	Denmark	133	Newborns	N/A	Immulite 2000	**5**	Authors selected the CP values based on data from a previous study	54
Kit et al, 2013 [55]	Cross-sectional (NHANES, 1988–2010)	Yes	USA	1988–1994: 248‡ 1999–2004: 336‡ 2005–2010: 392‡	4–11 yr	Non-Hispanic White, non-Hispanic Black, Mexican American or other	LC/MS	**0.05**	Assay LOD	55
Howrylak et al, 2014 [22]	Cohort Study, 2010–2011	Yes	USA	619	1–16 yr	White, African American, Multiracial or other	LC/MS	**0.1**	Assay LOD	22
Pino et al, 2004 [56]	Cross-sectional, 1995–1997	No	Chile	504	1 yr	N/A	RIA	**100**	Assay LOD (from a study conducted in 1987)	56
Mason et al, 2015 [57]	Cross-sectional (NHANES, 2007–2010)	No	USA	N/A†	≥ 3 yr	N/A	LC/MS	**0.05** **0.015**	Assay LOD	57
West et al, 2015 [23]	Cohort Study, 1980–2014	Yes	Finland	1,578‡	3–18 yr	N/A	GC	**3**	Authors selected the CP value based on data from a previous study	23
Merianos et al, 2017 [58]	Cross-sectional (NHANES, 2009–2012)	No	USA	2,707‡	3–11 yr	Non Hispanic White, non Hispanic-Black, Hispanic, other races/multiracial	LC/MS	**0.05**	Assay LOD	58
Yilmaz et al, 2018 [59]	Cross-sectional, 2012–2013	No	Turkey	150	1–3 yr	N/A	ELISA	**3**	Authors selected the CP value based on data from a previous study	59
Shenassa et al, 2017 [60]	Cross-sectional (NHANES, 1999–2012)	Yes	USA	2,679‡	3–5 yr	Non-Hispanic White, non-Hispanic Black, Mexican American or other	LC/MS	**10**	Authors used CP value based on data from a previous study	60
Hedengran et al, 2018 [24]	Cohort study, 2003–2004	Yes	Denmark	263	Newborns	N/A	LC/MS	**0.2**	Authors selected CP value based on data from a previous study	24
Nwosu et al, 2018 [61]	Cross-sectional (NHANES, 2009–2010)	No	USA	1,013≤	3–9 yr	Non-Hispanic White, Mexican American, Other Hispanics, African American or other	LC/MS	**0.05**	Assay LOD	61
Chelchowska et al, 2019 [25]	Cohort study, 2013–2015	Yes	Poland	80	Newborns	Caucasian	ELISA	**13.7**	Authors selected the CP value based on data from a previous study	25
Brody et al, 2019 [62]	Cross-sectional (NHANES, 2013–2016)	Yes	USA	2,833‡	3–11 yr	Non-Hispanic White, non-Hispanic Black, non-Hispanic Asian or Hispanic	LC/MS	**0.05**	Assay LOD	62
Biren et al, 2020 [63]	Cross-sectional (NHANES, 2015–2016)	Yes	USA	257	3–5 yr	Non-Hispanic white, non Hispanic-black, Mexican-American, other Hispanic or other/multirace.	LC/MS	**0.05**	Assay LOD	63
Rovio et al, 2020 [26]	Cohort study, 1980–2011	Yes	Findland	1,504‡	3–18 yr	N/A	LC/MS	**3**	Authors used CP value based on data from a previous study	26

Abreviations: N/A, not available; GLC/NPD, gas-liquid chromatographic procedure with nitrogen-phosphorus-specific detector; LOD, limit of detection; GC, gas chromatography; USA, United States of America; yr, year; RIA, radioimmunoassay; ROC, receiver operating characteristic; GC/MS, Gas- chromatography mass spectrometry; NHANES, National Health and Nutrition Examination Survey; LC/MS, isotope dilution-liquid chromatography-tandem mass spectrometry; CP, cut-point; SC, serum cotinine; SHS, secondhand smoke; ELISA, Enzyme-linked immunosorbent essay.

^†^Sample sizes includes children (3–11 yr), adolescents (12–19 yr) and adults (> 19 yr).

^‡^Sample size includes children aged 5 years and older.

Considering those studies assessing prenatal exposure, sample sizes ranged from 8 to 1,323 newborns. Among the 33 studies assessing postnatal exposure, 27 papers grouped children under 5 years with older/adolescents (aged 12–19) and 3 papers, also with adults (aged ≥ 20). The remaining 3, measured postnatal SHS exposure exclusively in children under 5 years with sample sizes ranging from 132 to 504 children [14,56,59] (Table 1 and Fig 2).

### Analytical technique, cut-point value/s, and method for cut-point identification

Isotope dilution liquid chromatography tandem mass spectrometry (LC-MS) was used in 35 of the selected papers followed by RIA in 7, GC/GC-MS/ GLC/NPD in 7 and ELISA in 2 (Fig 2). A total of 26 studies mentioned the assay LOD threshold as cut-point (Fig 2). In order to ascertain SHS exposure among children, cut-point values ranged between 0.015–35 ng/ml, excepting in one study that used 100 ng/ml; the most commonly used threshold was 0.05 ng/ml (17 out of 51) (Table 1). Eighteen articles selected the serum cotinine cut-point value/s based on previous studies (Fig 2). The lowest value was mentioned in North American studies and the highest, in a Chilean study [56].

One study calculated age-specific cut-point with receiver operating characteristic (ROC) curves [52]: 0.9 ng/ml (< 12 years) vs. 0.6 ng/ml (=/> 12 years). However, none of the selected articles proposed sex, race or age-specific serum cotinine cut-points in children under 5 years.

## Discussion

As summarized in Table 1, the cut-points to classify SHS exposure varied widely between studies. None of these studies have proposed age-specific cut-points to classify SHS exposure in children under 5 years. Only one study calculated two age-specific cut-points setting the reference age at 12. Commonly, SHS exposure thresholds were established attending to the assay LOD or based on previously defined adolescent and adult cut-points without accounting for population characteristics, analytical technique or prevalence of SHS exposure in the country of the study.

Several studies have concluded that cotinine metabolism is influenced by sex, race and age after having observed higher cotinine concentrations among children, females and non-Hispanic Blacks [42,44,64]. With respect to age, young children seem to have higher serum cotinine concentrations than older/adolescents and nonsmokers adults at similar exposures to SHS and this could be due to differences in cotinine metabolism and clearance. However, young children could also be more exposed to SHS due to faster respiratory rate, inability to distract themselves or breastfeeding by smoking mothers [42,64–66]. The NHANES, which has measured SHS exposure since 1988 by using questionnaires and serum cotinine, found higher cotinine concentrations among children aged 3–11 years compared to adolescents aged 12–19 years and adults [42,67].

In our systematic review, we have observed that serum cotinine cut-point values to characterize children’s exposure to SHS have varied remarkably since 1985 and across the countries, being the highest value mentioned in a Chilean study conducted in 1990s, more than 6,600 times that of the lowest used in US studies, 100 versus 0.015 ng/ml. European studies used values between 0.2–35 ng/ml. We only found three studies assessing postnatal exposure exclusively among children under 5 years, between 1–3 years, with disparate cut-points ranging from 2.5 ng/ml to 100 ng/ml.

We have observed that some of the studies that classified pre- or postnatal SHS exposure selected the threshold used to distinguish between smoker and non smoker (ranging from 3 to 20 ng/ml) and thus, being liable to have underestimate exposure on this target group. The only paper included in our review, which measured both pre- and postnatal exposure to SHS with serum cotinine, used the same cut-point for newborns and children aged 1 and 2 years [19].

The most widely used cut-point for the classification of prenatal SHS exposure, via newborns’ cord serum cotinine, was 1 ng/ml; One study obtained a cut-point of 1.78 ng/ml from the ROC curves to discriminate newborns from nonexposed and exposed nonsmokers with a sensitiviy and specificity of 60% [17]; this value exceeds some of those used to classify exposure among young children. However, taking into consideration the transfer of serum cotinine to fetus across the placenta and the higher metabolism and faster clearance of cotinine in pregnant women, it can not be disregarded that newborns may have lower cotinine concentrations than young children for the same exposure levels. Nonetheless, it should be acknowledged that the characterization of SHS prenatal exposure from cord serum cotinine samples from the newborn could be imprecise; taking into account that newborn refers to the moment of delivery and that the measured cotinine concentrations might cover the period of time in which women may be hospitalised [24,43].

In general terms, a decrease in the serum cotinine cut-point values has been observed over time and this could be partly explained by the better LOD threshold of the analytical techniques. Beginning in 2001–2002, the LC/MS assay LOD used to determine serum cotinine concentrations in the samples collected in NHANES was lowered in some studies from 0.05 to 0.015 ng/ml as a result of the introduction of a more sensitive mass spectrometer [42]. These two have been the LOD derived cut-points most widely used over time to classify exposure to SHS in the USA. Of note, it should be acknowledged that serum cotinine, like other biomarkers, has an important limitation, being that it does not provide information on the route and source of exposure. Although the main health risks associated with SHS are related to inhalation of fine particulate and gases from sidestream smoke [2,68], values around 0.015 ng/ml could reflect not only transient and accidental SHS exposure (sometimes unnoticed by children’s caregivers), but also third-hand smoke exposure (THS) or residual tobacco smoke (such as involuntary inhalation, ingestion or cutaneous absorption of nicotine particles from SHS deposited on surfaces, clothing or furniture which remain there long after smoke is gone) or exposure from other sources such as food. Thus, a child ingesting nicotine laden dust through crawling or hand-to-mouth activity (THS exposure) may have high serum cotinine from ingesting that dust, but could have experienced very little exposure to SHS. Previous studies reported that certain foodstuffs such as potatoes, tomatoes, eggplant, cauliflower, green peppers and black tea have measurable levels of nicotine [69,70]. Whilst food consumption levels of dietary nicotine are insignificant compared with moderate SHS exposure, the consumption of high quantities might contribute to low-level elevations in serum cotinine (e.g. 80 g of eggplant is equivalent to approximately 0.01 ng/ml of serum cotinine) [8]. Further studies in children could verify this hypothesis using detailed dietary information and sensitive cotinine biomarkers; this information could be useful to propose high level cut-points that could allow for a better characterization of the SHS exposure in children, differentiating significant exposure from minor or incidental exposure.

The lowering of the thresholds also results from the acknowledgement that no safe levels of SHS exposure can be established. Some prior investigators have emphasized that even very low levels of SHS exposure (serum cotinine concentrations < 0.1 ng/mL) can cause adverse effects on cognitive outcomes among children [71] and this could be also attributed to the higher lead concentrations found in children exposed to SHS compared to those unexposed [72]. In the USA, as a result of combined tobacco control policies, prevalence of tobacco consumption and SHS exposure have declined during the last 3 decades. Consequently, serum cotinine cut-points have become lower to be able to avoid misclassifying any SHS even at low level or accidental exposure [73]. Whilst these revised lower cut-points could be recommended from a health perspective, they could lead to an overestimation of the population exposure to SHS because as already mentioned, there is always a trade off between sensitivity and specificity. In this case, this lowering of the cut-point can lead to a higher false positive rate. This could impact on the calculations of SHS exposure in a given population and in term, on the estimated attributable burden of disease. The lack of consensus with respect to the most appropriate cut-point has also important implications with regards to the comparison of data and trend analysis.

This review has some limitations. First, we only used PubMed and EMBASE databases but we do not expect to have missed any relevant papers because we have expanded the search by including articles from the bibliography of those papers selected. Second, when limiting the search to English, Spanish, French and German, papers published in other languages might have been excluded. To the best of our knowledge, 1 paper published in Polish was excluded. Third, period of study was not especified in 4 cross-sectional studies [27,28,32,52]. Another limitation is the low sample size of some investigations with 3 articles without specification of the participants’ sample size [46,48,57]. Finally, 36 out of 51 studies were judged to be of low quality when applying the modified Newcastle-Ottawa scale, and the majority of the included studies grouped children under 5 years with older and adolescents, being this one the main limitation.

As the main strength, this is the first review on the serum cotinine cut-points used across different countries to assess SHS exposure in children. Prior investigations have identified the cotinine cut-point values used over time to discriminate between smokers and nonsmokers. The 2006 Surgeon General´s report mentioned the need to review the cut-points used when assessing tobacco consumption and SHS exposure among pregnant women considering the higher metabolic clearance rates and therefore, the shorter half-life of cotinine during pregnancy [1]. For those studies which found discrepancies between self-report and serum cotinine, most assumed that parents did not accurately report their child´s SHS exposure [15,18,53]. One study considered the possibility of misclassification due to the inadequacy of the cut-point [40]. A recent cohort study assessing SHS exposure among children younger than 5 years, observed that, compared to the assay LOD derived cut-point of 0.015 ng/ml, when using new age-specific cut-points, all of them higher than the mentioned assay threshold, concordance between maternal self-report and serum cotinine improved; actually, the percentage of children reclassified as SHS exposed with cotinine disminished in approximately fifty percentage points when using the age-specific cut-points obtained with ROC curves [74].

It is necessary to raise public awareness of the need to adopt 100% smoke-free policies worldwide aimed at avoiding the long-term adverse effects resulting from early exposures to tobacco smoke. SHS exposure has been associated with cognitive impairment and chronic diseases (such as respiratory and cardiovascular diseases) which begin early in childhood and cause premature disability, death and high healthcare costs. Thus, adequate classification of SHS exposure is warranted to accurately estimate the prevalence of SHS exposure and the attributable burden of disease to such exposure. Biomarker assessment of SHS provides an advantage over questionnaire assessment with respect to their accuracy; however, valid non-biased cotinine cut-points are needed. Considering that young children are especially vulnerable to the health consequences derived from SHS exposure, and the developmental changes in child behaviour, anatomy and physiology during the first years of life, further well-designed studies would be recommended to propose age-specific serum cotinine cut-points or multiple cut-point values which maximises both sensitivity and specificity to minimise misclassification either as exposed or unexposed based on cotinine concentrations.

## Conclusions

No studies have calculated serum cotinine age-specific cut-points to ascertained SHS exposure among children under 5 years old. The adverse health consequences derived from any level of SHS exposure, especially from the sidestream smoke, and the developmental changes in child behaviour, anatomy and physiology during the first years of life, support the need for age-specific cut-points for health research and public health purposes aimed at accurately estimating SHS exposure and attributable burden of disease to such exposure, and at reinforcing 100% smoke-free policies worldwide, both in homes, private vehicles and public places.

## Supporting information

S1 Checklist PRISMA checklist. (DOC)
